# Supplementary material for: Matrix Gla Protein drives stemness and tumor initiation in ovarian cancer
Source: Cell Death Dis. 2023 Mar 28;14(3):220. doi: 10.1038/s41419-023-05760-w (PMC10050398; doi:10.1038/s41419-023-05760-w)

Uncropped blots related to Figure 1D

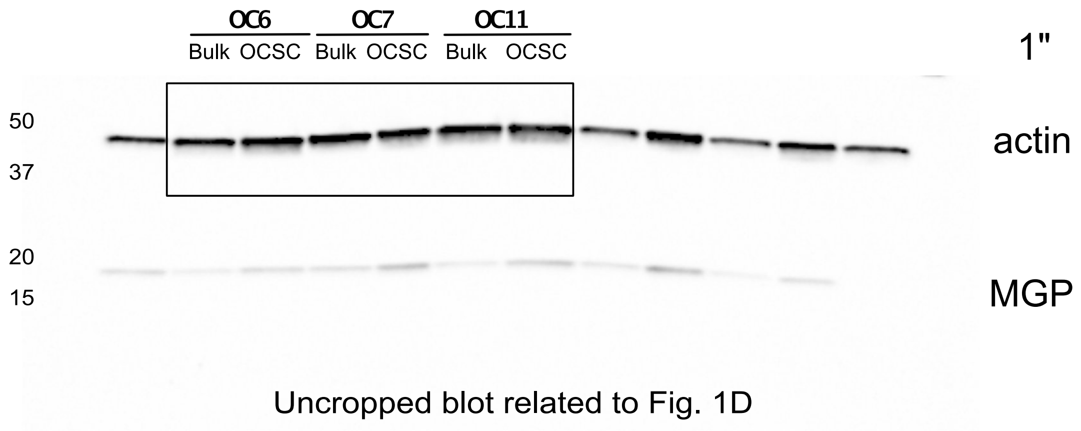

Uncropped blot related to Fig. 1D

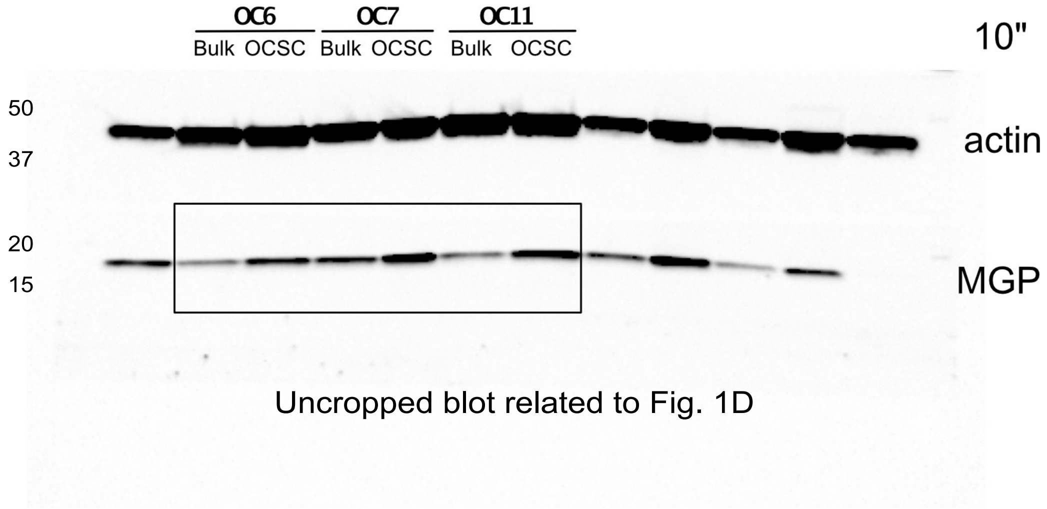

Uncropped blot related to Fig. 1D

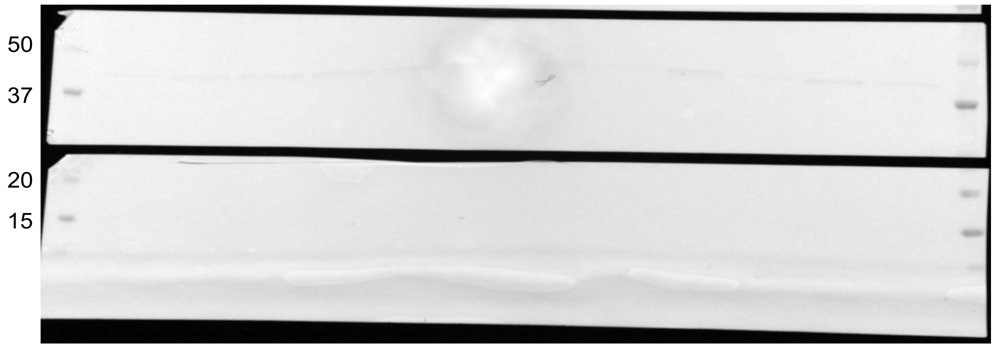

widefield image of pre-stained molecular weight markers

Uncropped blots related to Figure 7F

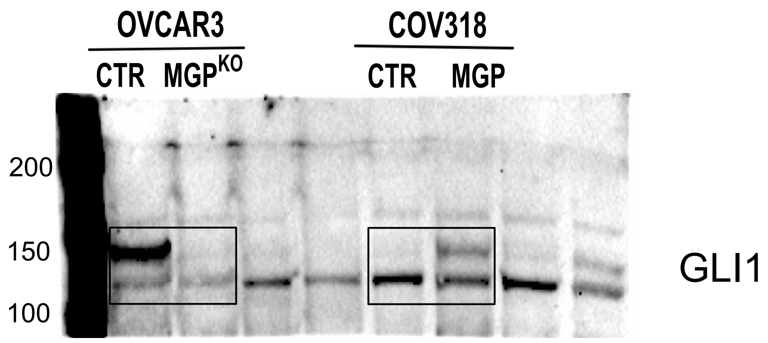

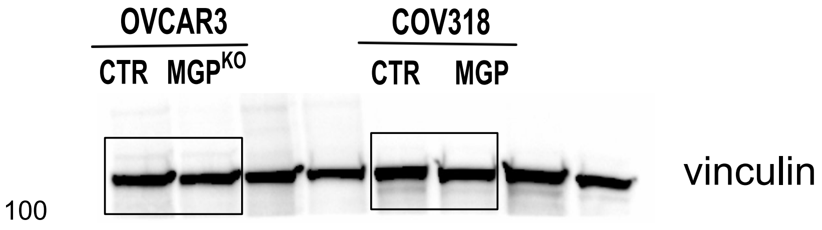

Uncropped blots related to Figure 7G

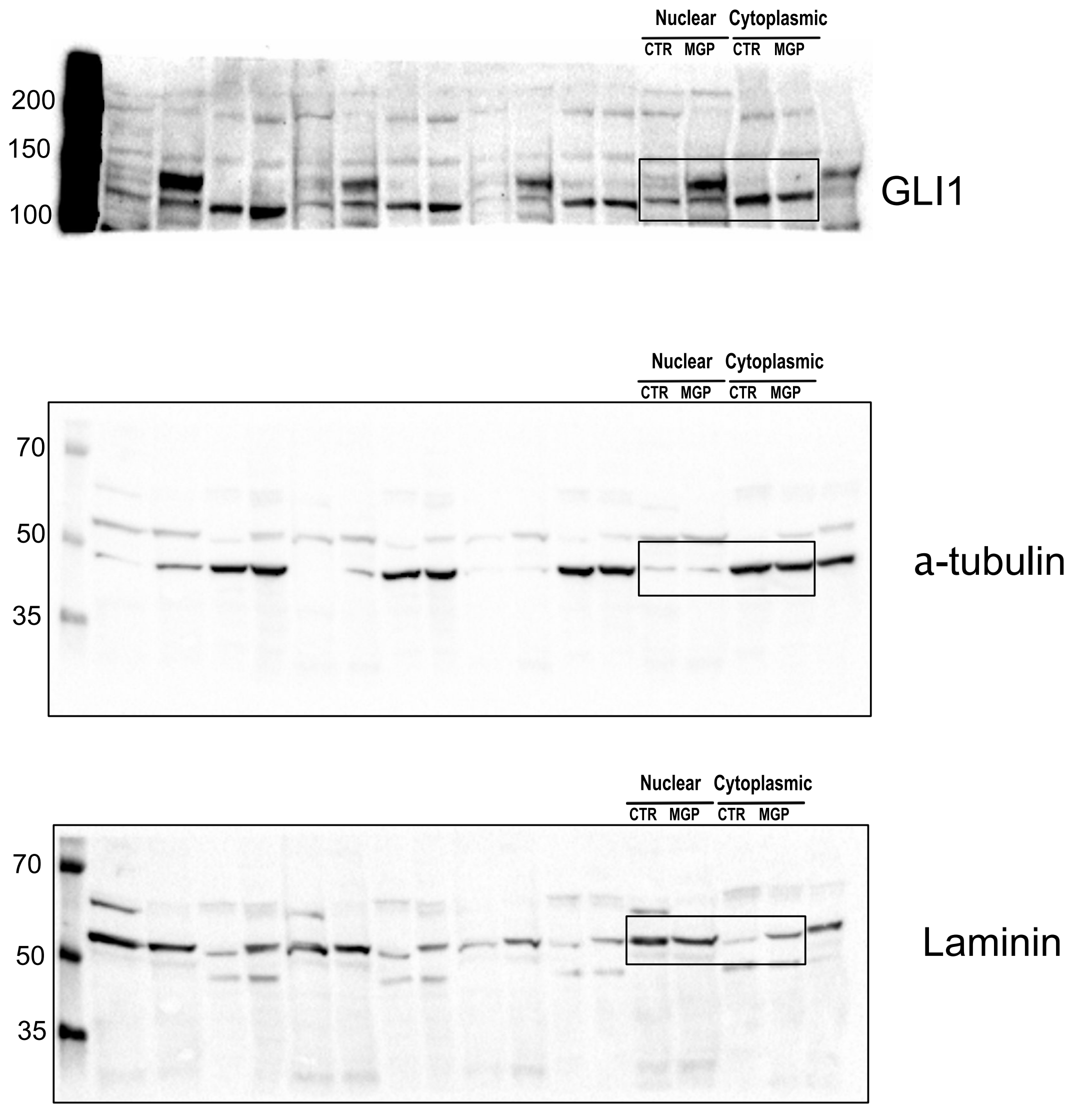

Uncropped blots related to Supplementary Figure 1B

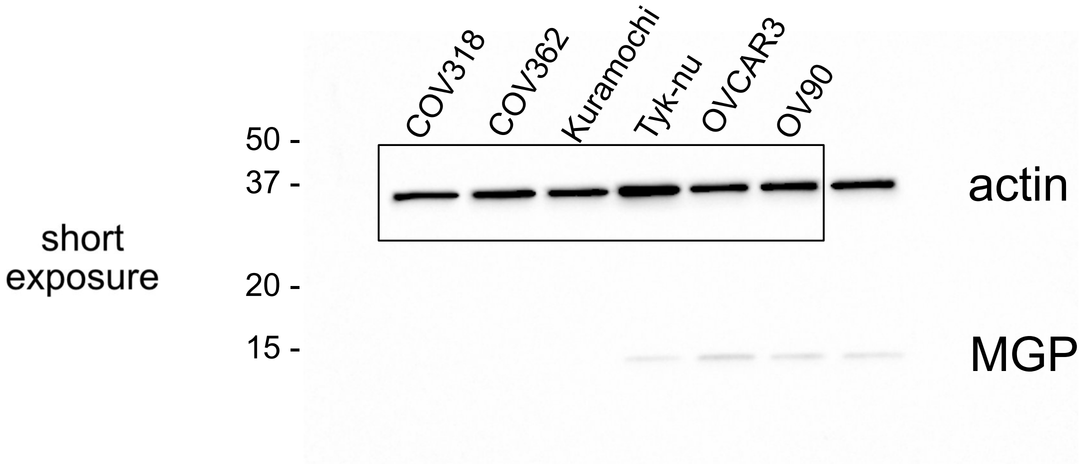

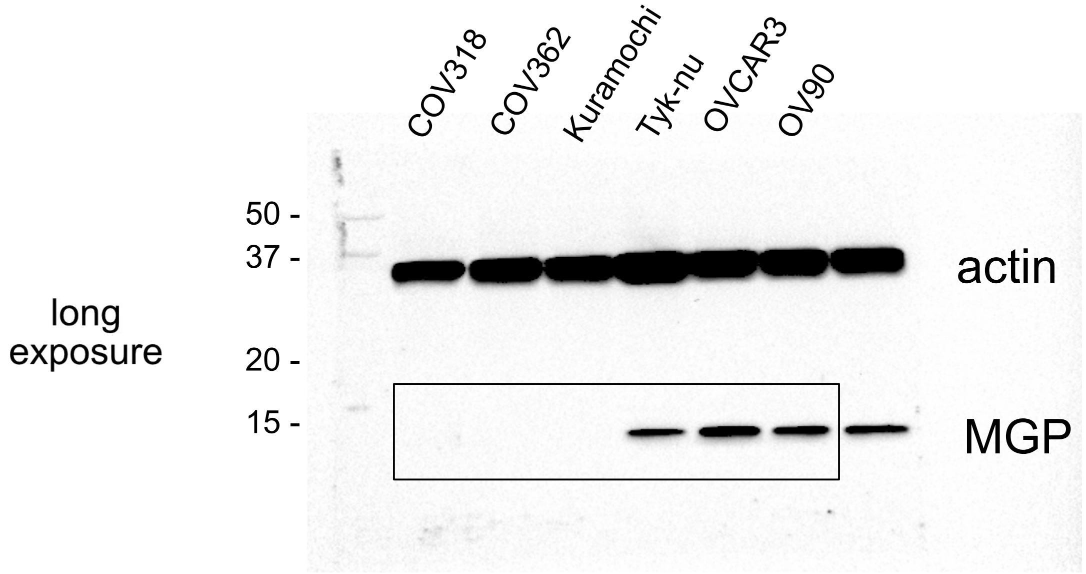

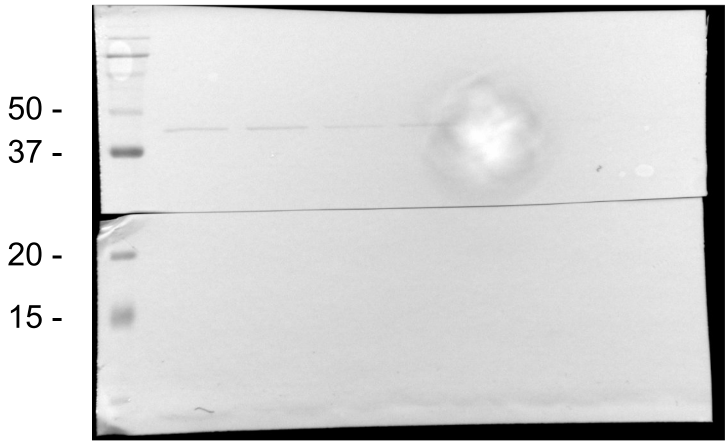

widefield image of pre-stained molecular weight markers

Uncropped blots related to Figure 1D

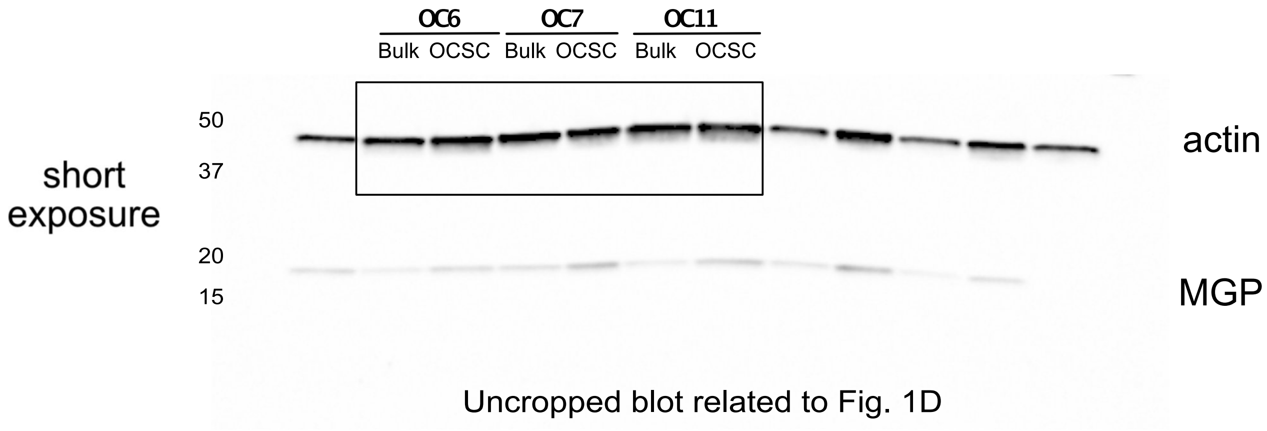

Uncropped blot related to Fig. 1D

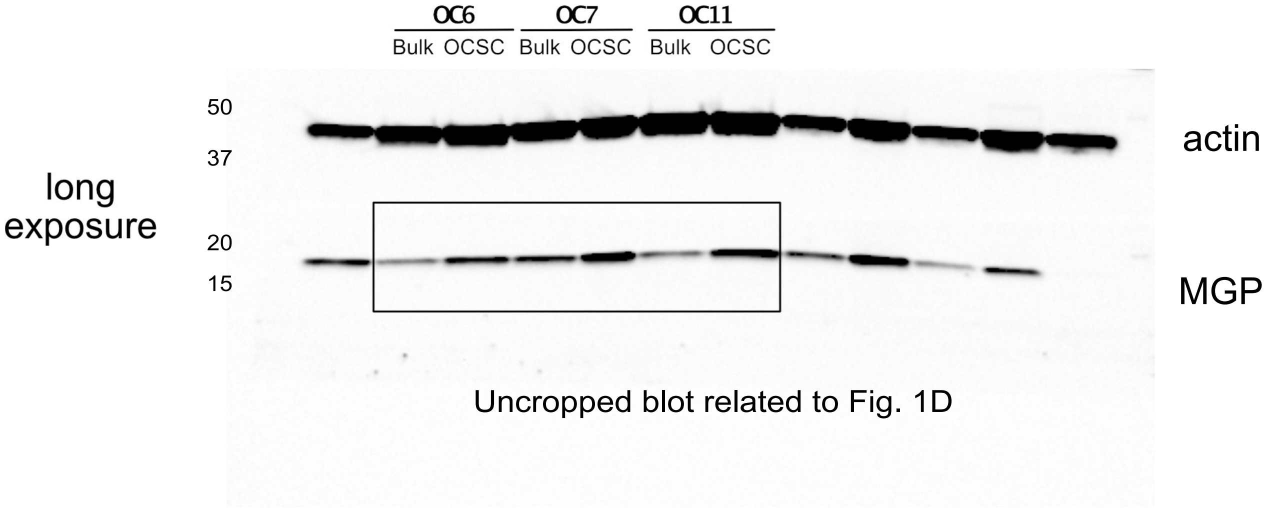

Uncropped blot related to Fig. 1D

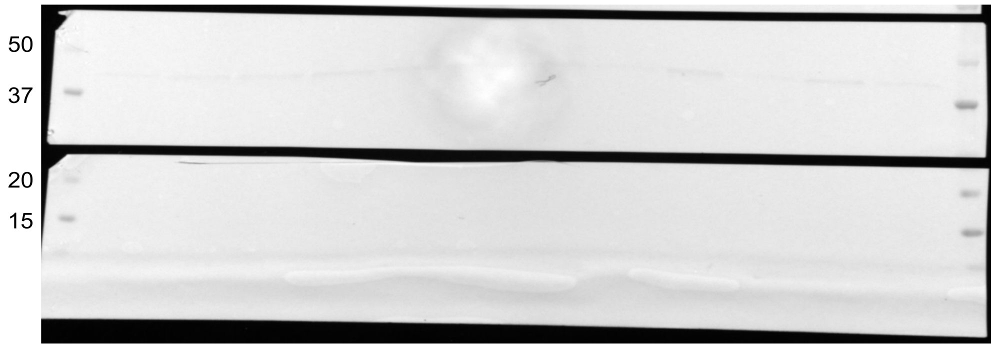

widefield image of pre-stained molecular weight markers

Uncropped blots related to Supplementary Figure 1G

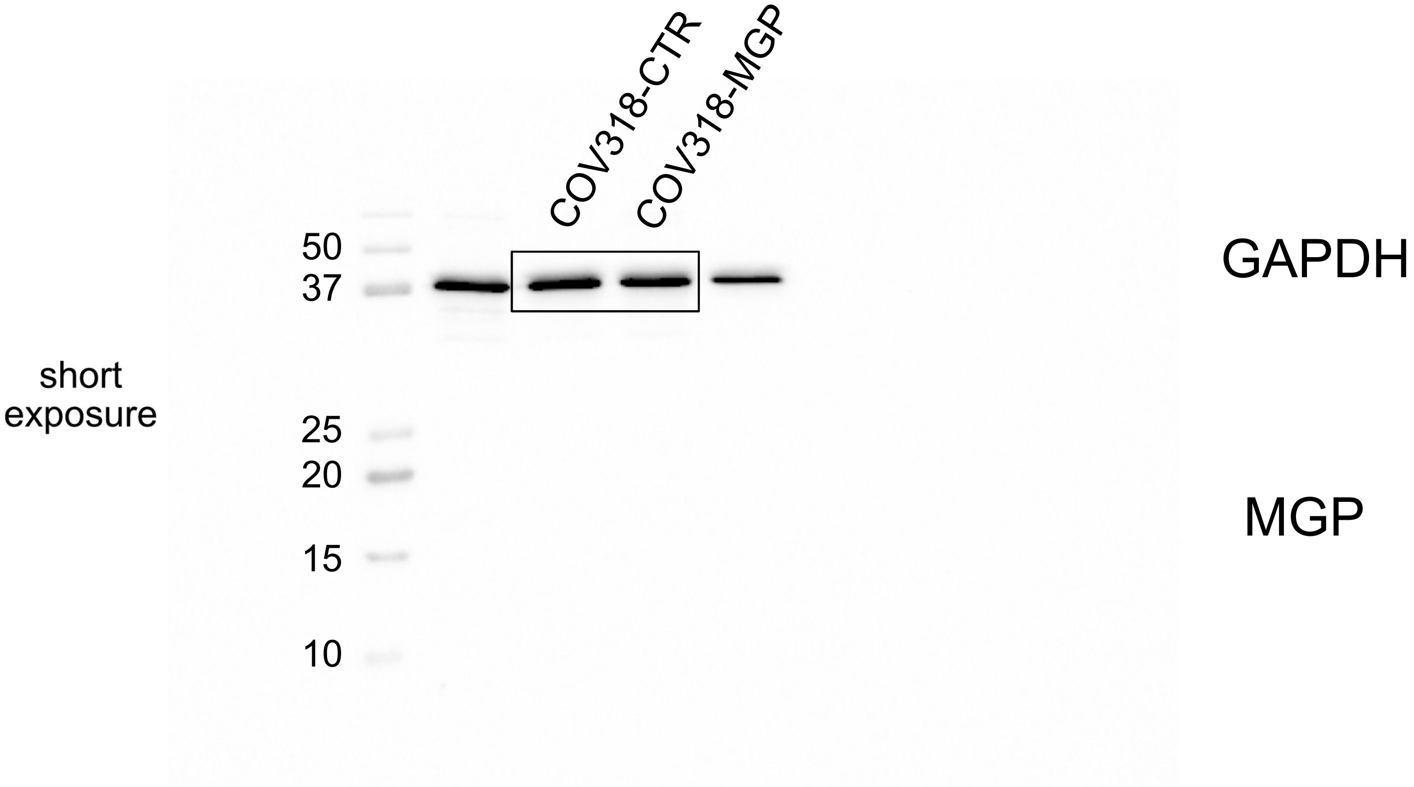

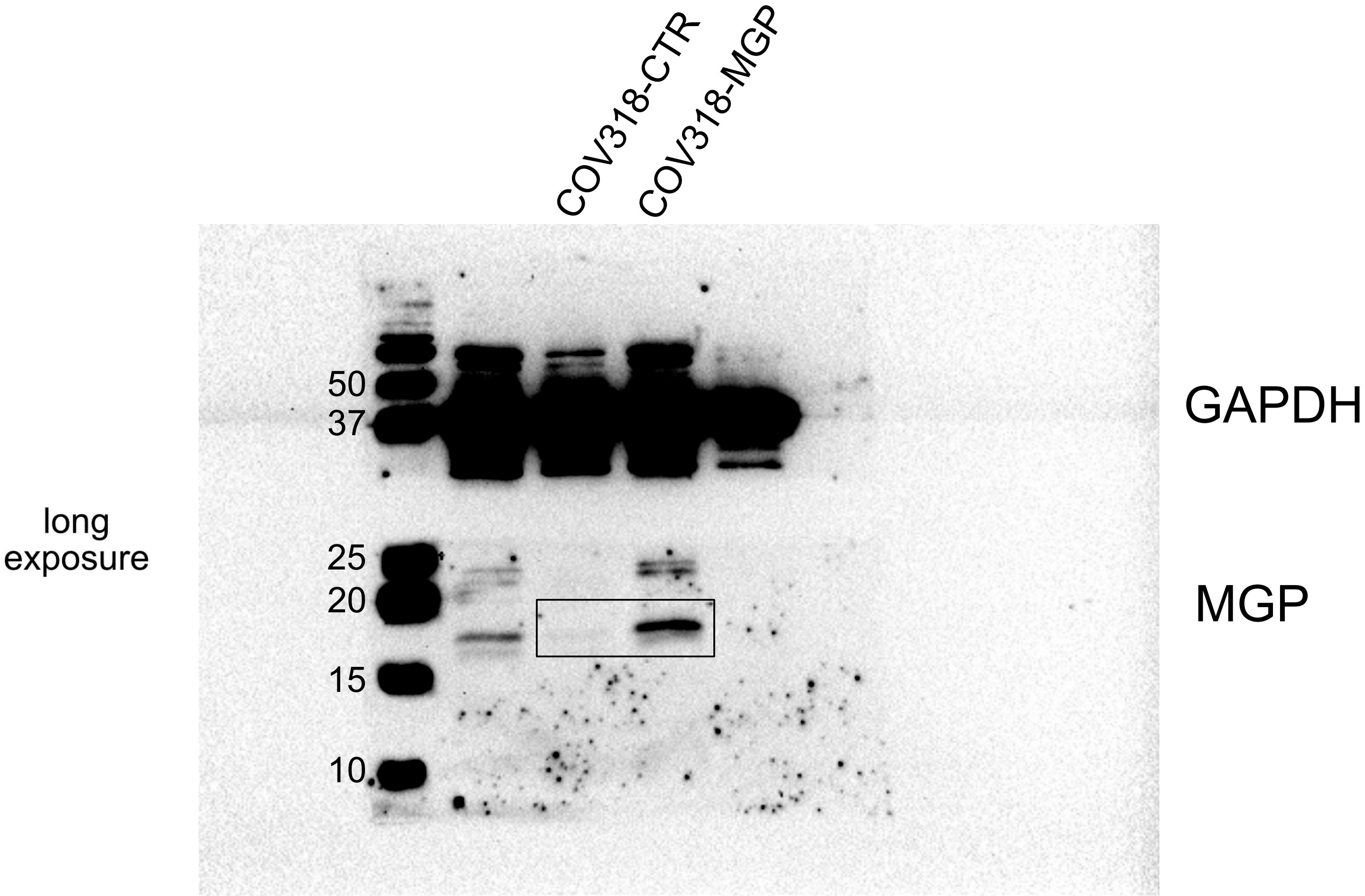

Supplement: Supplementary file 2 — Original Data File [file 41419_2023_5760_MOESM2_ESM.pdf]
